# Clinical and Imaging Findings in Childhood Posterior Reversible Encephalopathy Syndrome

**Published:** 2018

**Authors:** Serdal GUNGOR, Betul KILIC, Yilmaz TABEL, Ayse SELIMOGLU, Unsal OZGEN, Sezai YILMAZ

**Affiliations:** 1 Department of Pediatric Neurology, Faculty of Medicine, Inonu University, Malatya, Turkey.; 2 Department of Surgery, Faculty of Medicine, Inonu University, Malatya, Turkey.

**Keywords:** Children, Posterior reversible encephalopathy syndrome, Seizure, Atypical radiological findings

## Abstract

**Objective:**

Posterior reversible encephalopathy syndrome (PRES) is characterized by typical radiologic findings in the posterior regions of the cerebral hemispheres and cerebellum. The symptoms include headache, nausea, vomiting, visual disturbances, focal neurologic deficits, and seizures. The aim of this study is to evaluate the clinical and radiological features of PRES in children and to emphasize the recognition of atypical features.

**Materials & Methods:**

We retrospectively examined 23 children with PRES from Mar 2010-Apr 2015 in Inonu University Turgut Ozal Medical Center in Turkey. We compared the clinical features and cranial MRI findings between underlying diseases of PRES.

**Results:**

The most common precipitating factors were hypertension (78.2%) and medications, namely immunosuppressive and antineoplastic agents (60.8%). Manifestations included mental changes (100%), seizures (95.6%), headache (60.8%), and visual disturbances (21.7%) of mean 3.6 (range 1-10) days' duration. Cranial magnetic resonance imaging (MRI) showed bilateral occipital lesions in all patients, associated in 82.6% with less typical distribution of lesions in frontal, temporal or parietal lobes, cerebellum, corpus callosum, basal ganglia, thalamus, and brain stem. Frontal involvement was predominant, observed in 56.5% of patients. Clinical recovery was followed by radiologic resolution in all patients.

**Conclusion:**

PRES is often unsuspected by the clinician, thus radiologists may be the first to suggest this diagnosis on an MRI obtained for seizures or encephalopathy. Atypical MRI finding is seen quite often. Rapid diagnosis and treatment are required to avoid a devastating outcome.

## Introduction

Posterior reversible encephalopathy syndrome (PRES) is a clinico-radiological syndrome defined in 1996. Clinically, it is characterized by headache, changes in mental status, seizures, and visual disturbances (1). In many cases, severe headache is associated with a sudden rise in blood pressure (2). Except for hypertensive encephalopathy, etiology of PRES includes preeclampsia (3,4), sepsis (4), acute glomerulonephritis (5,6), lupus nephritis (7), organ transplantation (8), peritoneal dialysis (9), usage of cyclosporine A (1), tacrolimus (10,11), intrathecal methotrexate, cytarabine, daunorubicin, vincristine, and L-asparaginase (12). Although hypertension is frequent, 20%-30% are normotensive (10, 13). 

Pathogenesis is based on transient changes in the posterior circulation of the brain. Neuro-imaging is important for diagnosis. Computed tomography (CT) or magnetic resonance imaging (MRI) reveal diffuse edema more prominent in the parietal and occipital lobes bilaterally (4,14). Elimination of the underlying etiology and regulation of blood pressure are the first measures in PRES (8,13). 

Patients diagnosed and treated rapidly may recover completely within a few weeks. Due to the lack of specific clinical symptoms of the syndrome, it can be confused with other clinical diagnoses which may lead to unnecessary or inappropriate treatments (4,14). 

In this study, clinical and radiological findings of children diagnosed with PRES in our clinics were reviewed.

## Materials and Methods

We retrospectively analyzed the data of 23 pediatric patients who received the diagnosis of PRES from Mar 2010-Apr 2015 in various units of Inonu University Turgut Ozal Medical Center in Turkey: organ and bone marrow transplantation unit, Pediatric Intensive Care, Pediatric subspecialty departments of Gastroenterology, Oncology, Nephrology, or Rheumatology. Criteria for inclusion were 1) symptoms and signs compatible with PRES such as seizures, headache, visual disturbances, and altered mental function on a background of underlying primary disease, and 2) supportive imaging findings. Demographic data, clinical findings, neurological signs, length of hospital stay, concurrent medical illnesses, recently used drugs, blood pressure values during the symptoms, and cranial MRI findings were recorded. Hypertension was defined as systolic and/or diastolic blood pressure values higher than the 95% percentile for age (15).

MRI studies were performed on 1.5 Tesla MRI devices (Magnetom; Siemens, Germany). T2-weighted axial and sagittal, fluid-attenuated inversion recovery (FLAIR) axial, T1-weighted axial contrast-enhanced and unenhanced images were taken. Diffusion-weighted images and values were used and ADC maps were obtained. Cranial MRI was performed on all patients within the first three days after the onset of neurological symptoms. At least one more brain MRI was made during the follow-up. Two neuroradiologists interpreted all images. Typical MRI findings were defined as hyperintensity in subcortical white matter on T2A and FLAIR images and increase in ADC values in the posterior parietal, occipital, and temporal areas. Atypical MRI findings were defined as frontal lobe, brain stem, and cerebellar involvements, cytotoxic edema, hemorrhage, and contrast enhancement.

This study was performed with approval from the Institutional Review Board of Inonu University Research Council.

## Results

There were 23 children aged 1-16 (mean 7.8) yr, 13 boys, and 10 girls. Demographic and clinical data are summarized in Table 1. Among underlying primary diseases, malignancies (7/23), liver transplantation (7/23), and chronic renal diseases (3/23 chronic renal failure, 1/23 membranoproliferative glomerulonephritis, 1/23 lupus nephritis, 1/23 nephrotic syndrome), were most common (Table 1). Hypertension was the triggering factor in 78.2% of the cases, followed by immunosuppressive and antineoplastic agents (55.5%), liver transplantation (22.2%), and peritoneal dialysis (16.6%). Clinical signs suggesting the diagnosis of PRES: lethargy, confusion, coma, and encephalopathy were observed in all cases. Other clinical signs were seizures (95.6%), headache (60.8%), and visual impairment (21.7%). Seizure types were generalized (67.6%), partial (24.6%) or secondary generalized (7.8%).

**Figure 1:**
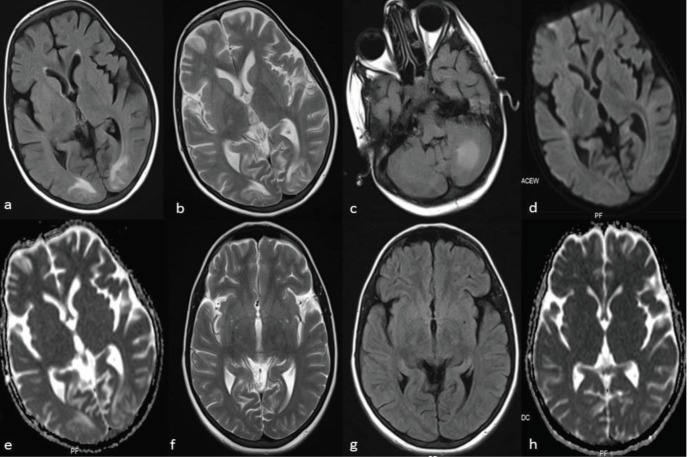
16 -year old girl after liver transplantation (patient 9). Axial MR images **a.** (T2-weighted) and **b. **and** c.** (fluid- attenuated inversion recovery) demonstrate edema in the occipital lobes and cerebellum. Diffusion-weighted images **d.** with b-1000 and **e.** with apparent diffusion coefficient (ADC) demonstrate increased diffusion in the occipital lobes. After 3 months of treatment, axial MR images **f**. and **g**. and ADC **h**. show normal findings

**Figure 2:**
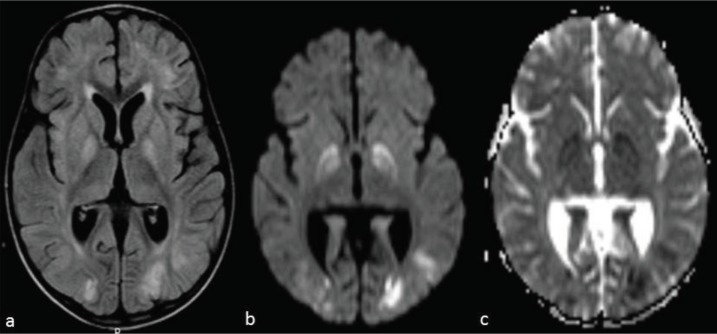
- year old boy after liver transplantation (patient 1). Axial MR image **a**. (fluid attenuation inversion recovery) demonstrates involvement of bilateral globus pallidi and frontal and occipital lobes. Diffusion-weighted images **b.** with b-1000 and **c.** with apparent diffusion coefficient demonstrate decreased diffusion in the occipital lobes and globus pallidus

Cranial imaging and EEG findings, clinical course, and antiepileptic drugs are summarized in Table 2. Cranial CT was performed in 16 patients: of these, four (25%) showed hypodense areas in posterior brain regions and hemispheres. Cranial MRI was performed at diagnosis in all, and on follow-up in 17 patients. Initial MRIs of all patients showed bilateral symmetric hyperintense lesions in occipital subcortical white matter on T2-weighted and FLAIR sequences, and hypointense lesions and vasogenic edema on T1-weighted images (Figures 1,2). In addition, frontal (13/23), parietal (12/23) and temporal (7/23) lobe involvements followed by cerebellum (6/23), basal ganglia (5/23), corpus callosum (7/23), brainstem (2/23), insular cortex (1/23), and thalamus (3/23) were observed. Lesions compatible with cytotoxic edema (11/23), contrast enhancement (1/23), and bleeding (3/23) were associated in certain cases (Table 2). 

Patients stayed in the pediatric intensive care unit for mean 16.2 (1-120) days. The mean duration of acute encephalopathy was 3.6 (1-10) days. Hypertensive patients were treated with nifedipine, amlodipine, verapamil, captopril, enalapril, furosemide, or losartan. Potential causative drugs such as tacrolimus, cyclosporine, cyclophosphamide, cisplatin, vincristine, L-asparaginase were discontinued. Eighteen patients had a follow-up cranial MRI performed within 2-12 wk (mean 37.6 d) after the first one. Partial resolution of lesions was observed in MRIs performed within the first three weeks, and complete resolution, on those performed at 4 wk or later. 

On long-term follow-up, one was lost to follow-up after discharge. Five patients died during the follow-up: the cause of death was neoplasm in three, and chronic liver disease with post-transplant complications in two. No patients died during the PRES episode. Overall, no correlation was detected between underlying disease and clinical or radiological findings. 

## Discussion

PRES may present with visual symptoms suggestive of posterior cerebral involvement, or with non-specific symptoms such as generalized or focal seizures, headache, nausea, vomiting, and mental changes. On the other hand, radiological findings are diagnostic. Morbidity and mortality in PRES may result from status epilepticus, intracranial hemorrhage or cerebral infarct (11,14,16,17). The pathophysiology of PRES is explained by two series of events initiated by hypertension: arterial spasm resulting in cytotoxic edema particularly in areas with limited arterial supply, and, more recently and more widely accepted, cerebral hyperperfusion and arterial hydrostatic edema followed by deterioration of cerebral autoregulation. In the 25% cases with normal blood pressure, the scenario is attributed to vasogenic edema due to various causes (8,11,18-20). On the other hand, the initiating role of hypertension can also be discussed, as hypertension can be secondary to acute encephalopathy and intracranial pressure. In our study, the predisposing factors were hypertension and antineoplastic or immunosuppressive medications. We had seven post-transplantation cases because our hospital is a transplantation referral center. The use of immunosuppressive agents after transplantation is a risk factor for PRES. It was detected in 4/40 patients with liver transplants (21). All our patients with organ transplantation had radiological findings other than typical posterior cerebral lesions. The presence of such radiological findings was not predictive of a specific course or outcome.

Our rate of atypical MRI findings, 82.6%, is higher than previously reported: 42.5% of 40 children and 43.8-58% in adult studies (22-25). Frontal lobe lesions were predominant. The susceptibility of the anterior cerebral regions to PRES as much as posterior ones may be related to the limitation of the anterior cerebral circulation in children, similar to the vertebro-basilar circulation (26). Because frontal involvement is frequent in any studies including those of the adult age group, the name of the syndrome as ‘posterior’ appears misleading. As previously reported, the extent of lesions were not correlated with the type and severity of clinical findings in our cases (4).

**Table 1:** Demographic Data, Clinical Characteristics, and Management of 23 Cases

Case	Age/Sex	Underlying Disorder	Clinical presentation	Precipitating Factors	Maximal BP (mm/Hg)	Antihypertensive agents
1	4/M	Acut liver failure, liver transplantation	Mental change, seizures	Tacrolimus, liver transplantation	100/60	_
2	9/M	Wilson disease, liver transplantation	Coma	Cyclosporin,hypertension, liver transplantation	145/85	Amlodipine
3	16/M	Lymphoblastic lymphoma	Mental change, seizures, headache	Vincristin, L-asparaginase	110/70	_
4	13/M	Chronic renal failure	Mental change, seizures, headache	Hypertension, Peritoneal dialysis	165/105	Captopril,peritoneal dialysis
5	7/F	Chronic renal failure	Mental change, seizures, headache, visual disturbance, papilloedema	Hypertension, Peritoneal dialysis	150/100	Enalapril,peritoneal dialysis
6	13/M	Membranoproliferative glomerulonephritis	Mental change, seizures, headache	Hypertension	185/125	Furosemide, nifedipine hemodialysis
7	9/F	WilmsTumor	Mental change, seizures, headache	Vincristine	110/65	_
8	11/M	Lupus Nephritis	Mental change, seizures,headache	Cyclophosphamide, hypertension	155/111	Hemodialysis,enalapril, losartan, furosemide
9	16/F	Chronic liver failure, liver transplantation	Mental change, seizures	Tacrolimus,liver transplantation, hypertension	135/95	Amlodipine
10	10/F	Acute lymphoblastic leukemia	Mental change, seizures,headache, visual disturbance, papilloedema	Vincristine, L-asparaginase, Hypertension	130/92	Verapamil
11	9/F	Chronic liver failure	Mental change, seizures, headache	Hypertension	140/100	Amlodipine, enalapril, furosemide
12	5/F	Chronic renal failure	Mental change, seizures, headache	Hypertension, Peritoneal dialysis,	145/100	Enalapril
13	6/M	Lymphoblastic lymphoma	Mental change, seizures, headache	Hypertension	160/121	Amlodipine, enalapril,losartan
14	9/F	Polyarteritis Nodosa	Mental change, seizures, headache	Hypertension	155/106	Verapamil
15	7/M	Nephrotic Syndrome	Mental change, seizures, headache	Hypertension	160/110	Enalapril, losartan
16	12/F	Wilson disease, liver transplantation	Mental change, seizures, visual disturbance	Tacrolimus, liver transplantation, hypertension	140/80	Verapamil
17	8/M	Chronic lymphoblastic leukemia	Mental change, seizures, visual disturbance,	Vincristine, L-asparaginase	140/87	Verapamil
18	6/M	Acute lymphoblastic leukemia	Mental change, seizures,visual disturbance,		130/90	
19	10/M	Inflamatory bowel disease	Mental change, seizures ,headache	Vincristine, L-asparaginase	185/110	Ramipril
20	1/M	Chronic liver failure, liver transplantation			95/55	Ramipril, losartan
21	3/F	Acute lymphoblastic leukemia	Mental change, seizures ,headache	Hypertension, total colectomy, tacrolimus	180/100	_
22	2/F	Chronic liver failure, liver transplantation	Mental change, seizures	Tacrolimus, liver transplantation	115/65	_
23	14/M	Chronic liver failure, liver transplantation	Mental change, seizures,headache	Fludarabine,	145/87	Verapamil,ramipril,losartan

**Table 2:** Neurologic Investigations and Outcames of 23 Cases

Case	Brain CT		Initial MRI findings			Follow up MRI (resolution/ interval)		Intensive care Unit staying time (day)		Recovery time		EEG	Antiepileptic therapy		Prognosis	
			Typical imaging	Atypical imaging												
1	None		Prt, Occ	Bg, Frt, cytotoxic edema hemorrhage		Subtotal (18 thday)		17		8		None	None		Diplegic cerebral palsy	
2	None		Prt, Occ	Frt, Cbl, hemorrhage, Bg, CC Bs, cytotoxicedema		None		10		-		None	None		Exitus	
3	Cerebral atrophy		Tmp, Occ	Frt, Cbl, CC		Total (1 month)		2		1		Normal	Levetiracetam		Exitus	
4	Normal		Prt, Occ	Frt, cytotoxic edema		Subtotal (15 thday)		7		3		None	Phenytoin		Recovery	
5	Hypodensities, brainedema		Prt, Occ	Frt, cytotoxic edema		None		7		2		Voltage suppression, generalized epileptic abnormalities	Sodyum Valproat		Recovery	
6	None		Prt, Occ	Frt, Cbl, cytotoxic edema		Subtotal (20 thday)		10		2		Normal	None		Recovery	
7	Normal		Tmp, Occ	None		Total (2 month)		2		2		None	None		Recovery	
8	None		Tmp, Occ	None		Subtotal (1 month)		3		3		Voltage suppression İsolated sharp waves	None		Recovery	
9	Cerebral atrophy		Prt, Occ	Frt, Cbl, CC		Total (2 month)		15		10		Normal	Levetiracetam		Recovery	
10	None		Occ	Cytotoxic edema, Gd+		Total (3 month)		7		3		Normal	Levetiracetam		Recovery	
11	Cerebral atrophy		Tmp, Occ	Frt, Cbl, Bg, Th		None		25		7		Normal	Levetiracetam		Follow-off	
13	Leftoccipital meningeal thickening		Prt,,Tmp,Occ	Frt, Cbl		Total (1 month)		2		2		Normal	Levetirasetam		Exitus	
14	None		Occ	Frt		Total (20 thday)		1		1		Normal	Levetiracetam		Recovery	
15	None		Prt, Occ	Frt		Subtotal (15 thday)		2		2		Voltage suppression	None		Recovery	
16	Cerebral atrophy		Tmp, Occ	Frt, CC, insular cortex, cytotoxic edema		Subtotal (15thday)		25		10		Voltage suppression, Left sharp waves	Levetiracetam,Topiramate		Recovery	
17		Cerebral atrophy	Prt, Occ		Th, CC, cytotoxic edema	Total (3 month)	4		2		Voltage suppression, Right sharp waves			Levetiracetam		Recovery	
18		Cerebral atrophy	Occ		None	Subtotal (20thday)	15		3		Normal			Levetiracetam		Recovery	
19		Hypodensities, brain edema	Prt, Occ		Th, cytotoxic edema	Subtotal (15 thday)	35		5		Voltage suppression, Left sharp waves			Sodyum Valproat		Recovery	
20		Normal	Prt,Occ		Frt, Th,CC, cytotoxic edema	Subtotal (1 month)	18		5		None			-		Recovery	
21		Cerebral atrophy	Prt, Occ		None	None	3		-		None			Phenytoin		Exitus	
22 23		Hypodensities, brain edema Cerebral and cerebellar atrophy	Prt, Occ Tmp,Occ		Bg,cytotoxic edema, hemorrhage Frt, Bg, Bs, CC, cytotoxic edema	Subtotal (1 month) None	120 30		5 2		Voltage suppression Voltage suppression			Levetiracetam Levetiracetam		Tetraplegia Exitus	

Occ, occipital; Prt, parietal; Th, thalamus; Tmp, temporal

Abbreviations: Bg, basal ganglia, Bs,brainstem; Cbl, cerebellum; CC, corpus callosum; CT, computed tomography; EEG, electroencephalography; Frt, frontal; Gd+, gadolinium enhancement;

There is no specific recommendation for the need and timing for follow-up MRI, but imaging findings may persist along with triggering factors (16). Lesions had resolved only partially in our patients (n=9) who had their follow-up MRI within the first month, and completely in the others imaged later. The association of cytotoxic edema was reported with a worse neurological outcome (25). We had twelve cases with cytotoxic edema, of which eight recovered completely. Two patients had motor deficits due to concomitant intracranial hemorrhage. 

Although this is not the largest series of pediatric PRES, its strengths lie in its single-center character, the inclusion of a considerable sub-series of transplant patients, the young age of patients (including two under 2 yr of age), and in particular, the uniform nature of imaging procedures, i.e., ADC mapping in all patients and evaluate by two neuroradiologists. Its limitations are the retrospective methodology and the different timing of follow-up images. 

**In conclusion,** early imaging of at-risk patients for PRES, longitudinal and frequent imaging during and after the episode of PRES would be of importance to establish the hemodynamic changes taking part in the pathogenesis. However, the critical clinical condition of these patients often precludes such studies, and retrospective evaluations continue to contribute to the pool of knowledge in this disorder. 

## Author`s contribution

Gungor S: Designed the study, management of patient, conducted laboratory tests, analyzed the data and revision of manuscript

Selimoglu A: Revised and approved the manuscript for important intellectual content of the paper

Kilic B: Diagnosis, management and writing the manuscript 

Tabel Y: Management of patient, conducted laboratory tests

Ozgen U: Supervision of the work and revision of manuscript.

Yilmaz S: Management of patient, conducted laboratory tests

Sıgırcı A: Conducted laboratory tests and analyzed the data

All authors agreed to be accountable for all aspects of the work in ensuring that questions related to the accuracy or integrity of any part of the work are appropriately investigated and resolved.

## Conflict of interest

Authors declare they have no conflict of interest.
